# Identification of 11(13)-dehydroivaxillin as a potent therapeutic agent against non-Hodgkin's lymphoma

**DOI:** 10.1038/cddis.2017.442

**Published:** 2017-09-14

**Authors:** Xinhua Xiao, Huiliang Li, Huizi Jin, Jin Jin, Miao Yu, Chunmin Ma, Yin Tong, Li Zhou, Hu Lei, Hanzhang Xu, Weidong Zhang, Wei Liu, Yingli Wu

**Affiliations:** 1 Shanghai General Hospital, Shanghai Jiao Tong University School of Medicine, Shanghai 200080, China; 2 Hongqiao International Institute of Medicine, Shanghai Tongren Hospital/Faculty of Basic Medicine, Chemical Biology Division of Shanghai Universities E-Institutes, Key Laboratory of Cell Differentiation and Apoptosis of the Chinese Ministry of Education, Shanghai Jiao Tong University School of Medicine, Shanghai 200025, China; 3 School of Pharmacy, Second Military Medical University, Shanghai 200433, China; 4 School of Pharmacy, Shanghai JiaoTong University, Shanghai 200240, China; 5 Department of Hematology, Shanghai Ruijin Hospital, Shanghai Jiao Tong University School of Medicine, Shanghai 200025, China

## Abstract

Despite great advancements in the treatment of non-Hodgkin lymphoma (NHL), sensitivity of different subtypes to therapy varies. Targeting the aberrant activation NF-*κ*B signaling pathways in lymphoid malignancies is a promising strategy. Here, we report that 11(13)-dehydroivaxillin (DHI), a natural compound isolated from the Carpesium genus, induces growth inhibition and apoptosis of NHL cells. Multiple signaling cascades are influenced by DHI in NHL cells. PI3K/AKT and ERK are activated or inhibited in a cell type dependent manner, whereas NF-*κ*B signaling pathway was inhibited in all the NHL cells tested. Applying the cellular thermal shift assay, we further demonstrated that DHI directly interacts with IKK*α*/IKK*β* in NHL cells. Interestingly, DHI treatment also reduced the IKK*α*/IKK*β* protein level in NHL cells. Consistent with this finding, knockdown of IKK*α*/IKK*β* inhibits cell proliferation and enhances DHI-induced proliferation inhibition. Overexpression of p65, p52 or RelB partially reverses DHI-induced cell growth inhibition. Furthermore, DHI treatment significantly inhibits the growth of NHL cell xenografts. In conclusion, we demonstrate that DHI exerts anti-NHL effect *in vitro* and *in vivo*, through a cumulative effect on NF-*κ*B and other pathways. DHI may serve as a promising lead compound for the therapy of NHL.

Non-Hodgkin lymphoma (NHL), a heterogeneous group of lymphoid malignancies, is the most common hematologic malignancy in the world,^1, 2^ ranking 6th in the incidence of cancer. The death rates of NHL for females and males were 8th and 9th, respectively. NHL consists of various types, such as the diffuse large B-cell lymphoma (DLBCL) and Burkitt lymphoma (BL). DLBCL is the most common type of NHL, accounting for 30–40% of newly diagnosed patients in the USA.^3^ DLBCL is also comprised of distinct subtypes, including activated B-cell-like DLBCL (ABC-DLBCL) and germinal center B-cell-like DLBCL (GCB-DLBCL).^4^ The prognosis of the ABC subtype of DLBCL is worse than the GCB subtype due to the constitutive activation of NF-*κ*B.^5^ BL, on the other hand, is derived from the germinal center^6^ and generally associated with Epstein–Barr virus infection and c-Myc chromosomal translocations, which lead to constitutive activation of c-Myc.^7^ Conventional treatment for NHL consists of CHOP (cyclophosphamide, doxorubicin, vincristine and cytarabine) followed by radiotherapy.^8^ As Rituximab, a monoclonal antibody against CD20, was approved for use, the clinical outcomes and survival in patients with NHL has improved, especially when combined with CHOP.^9^ Unfortunately, side-effects including cardiac arrhythmias, hypotension and renal failure occur during therapy. Furthermore, a considerable percentage of patients relapse during treatment. Therefore, developing more efficient and safer chemotherapeutic agents to improve the overall outcomes for NHL is required.

NF-*κ*B is a nuclear transcription factor that regulates cell proliferation and survival in lymphocytes.^10^ In rest cells, the NF-*κ*B complexes are sequestered in the cytoplasm by a variety of inhibitor proteins such as I*κ*B*α*, I*κ*B*β* and I*κ*Bγ. When the NF-*κ*B complexes are activated by different stimuli, such as TNF*α* and LPS, I*κ*B*α* is phosphorylated by I*κ*B kinase complex (IKK) and subsequently degraded by the proteasome. NF-*κ*B is then released from I*κ*B*α* and translocated to the nucleus, where it regulates gene expression. Constitutively activated NF-*κ*B pathway protects the malignant cells from apoptosis and confers resistance to chemotherapy.^11, 12^ Aberrant NF-*κ*B activation is noted in various human lymphoma malignances,^13, 14, 15, 16^ including DLBCL and BL.^17, 18, 19^ Thus, NF-*κ*B is a potential target for anti-lymphoid malignancy drug discovery.

11(13)-dehydroivaxillin (DHI) is a natural compound that can be extracted from the Carpesium genus, a plant used as an anti-pyretic, analgesic and febrifuge in traditional Chinese medicine.^20^ DHI also demonstrates efficient antiplasmodial activity *in vivo*.^21^ In this study, we report that DHI exhibits strong anti-NHL activity *in vitro* and *in vivo*. The mechanisms studies have revealed that inhibition of IKK*α/β* and other survival pathways such as AKT and ERK are involved in the anti-NHL effect of DHI. DHI represents a promising lead compound for the treatment of NHL.

## Results

### DHI inhibits proliferation and reduces viability of human NHL cells

**Figure 1:**
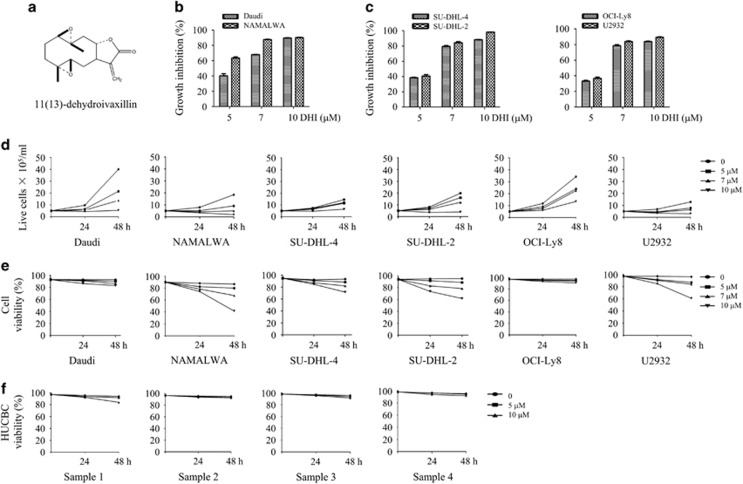
The effect of DHI on NHL cells (**a**, **b** and **c**). Chemical structure of DHI (**a**). Burkitt lymphoma cells, Daudi and NAMALWA (**b**), Diffuse large B-cell lymphoma cells, SU-DHL-4, SU-DHL-2, OCI-Ly8 and U2932 cells (**c**) were treated with various concentrations of DHI (0, 5, 7, 10 *μ*M) for 24 h. The inhibition of proliferation was evaluated by CCK-8 assays. (**d** and **e**) Daudi, NAMALWA cells and SU-DHL-4, SU-DHL-2, OCI-Ly8 and U2932 cells were plated at a density of 5 × 10^5^ cells/ml and then exposed to different concentrations of DHI for 24 and 48 h. The living cell number or cell viability was evaluated by trypan blue exclusion assay. (**f**) Human mononuclear cells separated from umbilical cord blood cells were treated with increasing concentrations of DHI for 24 and 48 h and subjected to trypan blue exclusion assay to determine cell viability. Data are representative of three or more experiments with similar results. All values represent the means±S.D. of three independent experiments. *P<0.05; **P<0.01 *versus* the control

To evaluate the effect of DHI (Figure 1a) on the proliferation of NHL, BL cells – Daudi and NAMALWA cells – and DLBCL cells – SU-DHL-4 (GCB-DLBCL), SU-DHL-2 (ABC-DLBCL), OCI-Ly8 (GCB-DLBCL) and U2932 (ABC-DLBCL) were treated with various concentrations of DHI (0, 5, 7, 10 *μ*M) for 24 h. Cell proliferation was evaluated by CCK-8 assay. DHI exhibited inhibitory effect on these lymphoma cells (Figures 1b and c). Moreover, cell viability was determined by the trypan blue exclusion assay. DHI reduced cell viability and inhibited the cell proliferation in some NHL cell lines in a dose- and time-dependent manner. Among them, NAMALWA, U2932 and SU-DHL-2 cells were considered the most sensitive to DHI-induced proliferation inhibition and cell death (Figures 1d and e). However, DHI did not affect the viability of mononuclear cells isolated from umbilical cord blood cells (Figure 1f). Together, these data suggest that DHI selectively inhibits the growth of lymphoma cells.

### DHI induces apoptosis in NHL cells

**Figure 2:**
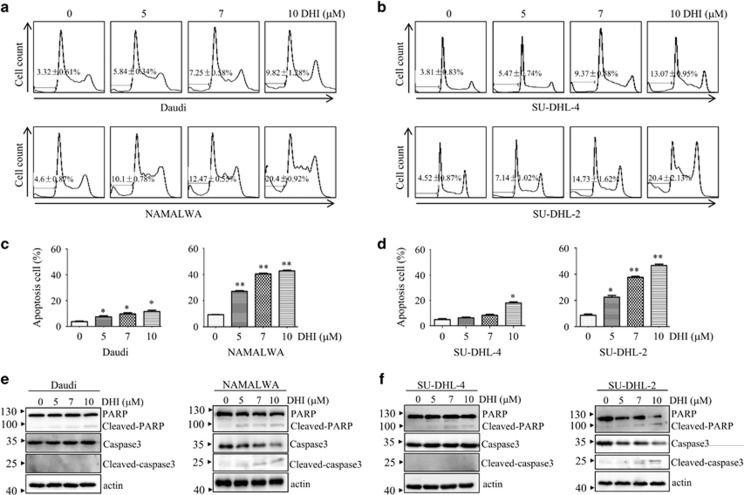
DHI induces apoptosis of NHL cells. (**a** and **b**) Effects of DHI at various concentrations on the cell cycle distribution of Daudi, NAMALWA cells (**a**) and SU-DHL-4 and SU-DHL-2 cells (**b**) treated for 24 h. (**c** and **d**). NHL cells were treated with different concentrations of DHI for 24 h. Annexin V positive Daudi and NAMALWA cells (**c**), SU-DHL-4 and SU-DHL-2 cells (**d**) were examined by flow cytometry. All values represent the means±S.D. of three independent experiments. *P<0.05; **P<0.01 *versus* the control. (**e** and **f**) NHL cells were treated with the indicated concentrations of DHI for 24 h, followed by western blotting for the indicated proteins

To investigate whether DHI induces apoptosis in NHL cells, Daudi, NAMALWA, SU-DHL-4 and SU-DHL-2 cells were exposed to various concentrations of DHI for 24 h. Cell population in the subG1 phase was examined by flow cytometry. In all the cell lines tested, DHI treatment induced an increase of the cell population in the subG1 phase to varying degrees (Figures 2a and b). In contrast to other cells, S phase arrest was observed in DHI-treated NAMALWA cells, which was accompanied by the reduction of cyclin A expression (Supplementary Figure S1). The apoptotic induction effect of DHI was further evaluated by Annexin V/PI staining using flow cytometry. The results demonstrated that NAMALWA and SU-DHL-2 are more sensitive than Daudi and SU-DHL-4 cells to DHI-induced apoptosis (Figures 2c and d). Consistent with these observations, DHI treatment induced cleavage of caspase-3 and PARP in NAMALWA and SU-DHL-2 cells, but not in Daudi and SU-DHL-4 cells (Figure 2e and f). These results indicate that DHI induces apoptosis in the treated lymphoma cells.

### DHI suppresses the NF-*κ*B transcriptional activity

**Figure 3:**
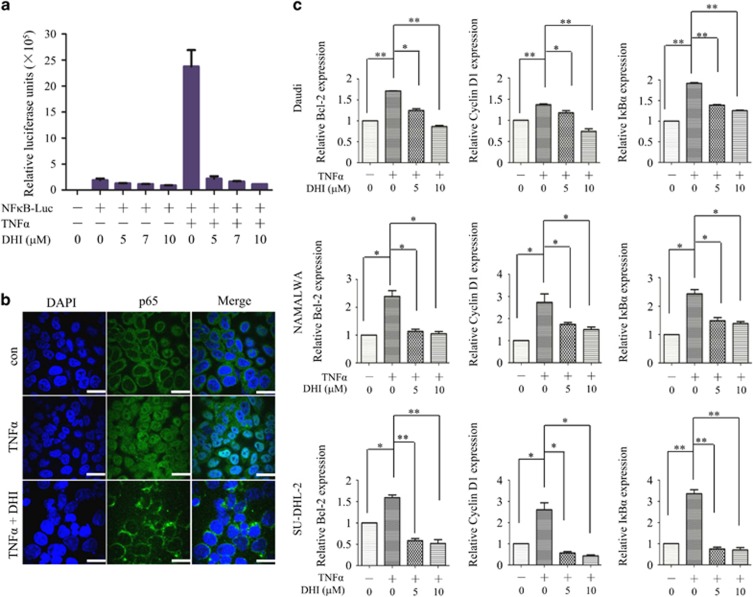
DHI inhibits NF-*κ*B activation induced by TNF*α*. (**a**) Daudi cells were stably transfected with NF-*κ*B luciferase reporter plasmid. An equivalent number of cells were plated in 24-well plates and treated with 0, 5, 7 or 10 *μ*M of DHI for 4 h, followed by treatment with or without TNF*α* (15 ng/ml) for 4 h. Luciferase activity was measured using Bright-Glo reagents (Promega). (**b**) HeLa cells were treated with or without the indicated concentrations of DHI for 12 h, followed by stimulation with or without TNF*α* (15 ng/ml) for 30 min. Immunofluorescent staining of NF-*κ*B p65 was then performed and assayed by confocol microscopy. Green signaling represents p65 staining, whereas blue signaling indicates nuclei stained with DAPI. Scale bar, 20 *μ*m. (**c**) Daudi, NAMALWA, and SU-DHL-2 cells were pre-treated with or without 5, 10 *μ*M DHI for 6 h, followed by stimulation with TNF*α* (15 ng/ml) for 90 min. qRT-PCR was then used to detect the indicated mRNA. Data are representative of three or more experiments with similar results. All values represent the means±S.D. of three independent experiments. *P<0.05; **P<0.01 *versus* the control

Given the important role of NF-*κ*B pathway in the survival of lymphoma cells,^11, 12^ we hypothesized that DHI may affect NF-*κ*B activity. To test this hypothesis, Daudi cells stably transfected with NF-κB-dependent luciferase reporter were treated with DHI in the presence or absence of 15 ng/ml of TNF*α*. As shown in Figure 3a, DHI significantly inhibited TNF*α*-induced NF-κB activation. Consistent with this result, the immunostaining assay and nuclear and cytoplasmic extraction assay showed that DHI inhibited nuclear translocation of NF-*κ*B p65 in HeLa cells (Figure 3b) and SU-DHL-2 cells (Supplementary Figures S2a and b) but did not affect the nuclear localization of p50 (Supplementary Figure S3). Moreover, TNF*α*-induced upregulation of NF-*κ*B p65 target genes,^22^ such as cyclin D1, Bcl-2 and IκB*α*, were also suppressed by DHI treatment in Daudi, NAMALWA and SU-DHL-2 cells (Figure 3c). In addition to NF-*κ*B, we also tested other signaling pathways downstream of the B-cell receptor (BCR) that have been shown to have pivotal role in B-cell survival, such as the PI3K/AKT and ERK pathways.^23, 24^ In response to DHI, both activation and inhibition of AKT and ERK could be induced in different cell lines. For example, DHI inhibited the phosphorylation of AKT and ERK in Daudi, NAMALWA and U2932 cells, increased phosphorylation of AKT but decreased phosphorylation of ERK in SU-DHL-4 cells, and increased phosphorylation of AKT and ERK in SU-DHL-2 cells. DHI had no effect on the phosphorylation of AKT and ERK in OCI-Ly8 cells (Supplementary Figure S4a). Taken together, these results indicate that DHI affects PI3K/AKT and ERK pathways in a cell-type-dependent manner and the common pathway affected by DHI in NHL cells is the NF-*κ*B signaling pathway.

### DHI suppresses IKK activation

**Figure 4:**
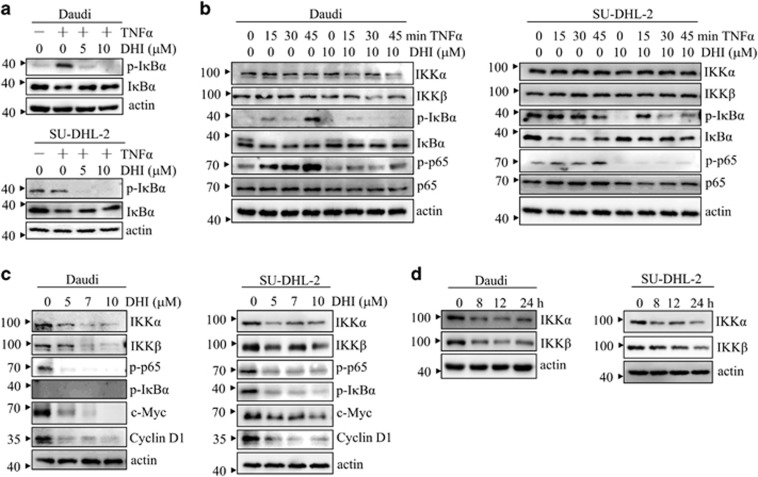
DHI suppresses the NF-*κ*B signaling cascade. (**a**) Daudi and SU-DHL-2 cells were pre-treated with DHI at 0, 5, or 10 *μ*M for 4 h, followed by stimulation with TNF*α* (15 ng/ml) for 30 min. Expression of p-I*κ*B*α* and I*κ*B*α* in the whole cell lysate was then analyzed. *β*-actin was used as loading control. (**b**) Daudi and SU-DHL-2 cells were treated with DHI (10 *μ*M) for 4 h and then exposed to TNF*α* (15 ng/ml) for varying time intervals. Whole cell lysates were then prepared for NF-*κ*B analysis using specific antibodies. (**c**) Daudi and SU-DHL-2 cells were treated with DHI (0, 5, 7, 10 *μ*M) for 24 h and analyzed by western blotting against the specific antibodies as indicated. (**d**) After exposure to DHI (10 *μ*M) for various times, the indicated proteins from Daudi and SU-DHL-2 cells were detected via western blotting. All experiments were repeated three times with the same results

NF-*κ*B nuclear translocation is controlled by the inhibitory I*κ*B*α* proteins. Phosphorylation of I*κ*B*α* by IKK leads to its proteasomal degradation, thereby allowing nuclear translocation of NF-*κ*B.^25^ Given that DHI inhibits NF-*κ*B p65 nuclear translocation, we hypothesized that DHI may do so by inhibiting the IKK/I*κ*B*α* signaling pathway. To test this hypothesis, Daudi, NAMALWA and SU-DHL-2 cells were pre-treated with various concentrations of DHI for 4 h followed by TNF*α* stimulation. Western blotting results showed that TNF*α*-induced I*κ*B*α* phosphorylation and degradation could be blocked by DHI (Figure 4a and Supplementary Figure S5a). DHI also inhibited LPS-induced I*κ*B*α* phosphorylation and degradation (Supplementary Figure S5b). Moreover, time course experiments demonstrated that pre-treatment with DHI for 4 h could effectively block the phosphorylation of I*κ*B*α* and p65 in Daudi and SU-DHL-2 cells (Figure 4b). Treatment with various doses of DHI for 24 h markedly reduced the protein level of IKK*α/β* and p-I*κ*B*α* in Daudi and SU-DHL-2 cells (Figure 4c). c-Myc and cyclin D1, two NF-*κ*B gene targets essential for lymphoma cell proliferation and survival,^26, 27^ were also dramatically decreased by DHI treatment (Figure 4c). Reduction of IKK*α/β* could be observed as early as 8 h (Figure 4d). These results indicate that DHI blocks NF-*κ*B activation by inhibiting the IKK/I*κ*B*α* signaling pathway.

### DHI interacts with IKK*α/β* and IKK*α/β* knockdown enhances the effect of DHI in NHL cells

**Figure 5:**
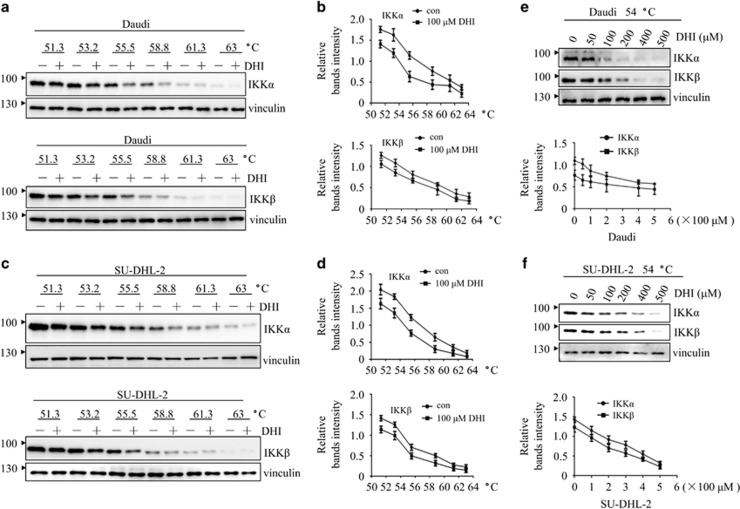
DHI interacts with IKK*α/β*. The effect of DHI on thermal stabilization of IKK*α/β* in Daudi and SU-DHL-2 cells, using various temperatures (**a** and **c**) and various dosages (**e** and **f**), were analyzed by western blotting. The density of the IKK*α/β* bands were quantified by quantity one software (**b**, **d** and **e**, **f**). All experiments were repeated three times with the same results

In order to investigate whether the DHI-induced inhibition of NF-*κ*B activation was due to a direct interaction with IKK*α/β*, a cellular thermal shift assay (CETSA) experiment was conducted. CETSA is a newly developed method for evaluating drug-binding to target proteins in cells and tissue samples.^28, 29^ As shown in Figures 5a and d, compared with DMSO, addition of DHI reduced thermal stability of IKK*α/β* protein in Daudi cells and SU-DHL-2 cells. Moreover, increasing the concentration of DHI decreased the thermal stability of IKK*α/β* proteins (Figure 5e and f). As a negative control, we evaluated the thermal stability of vinculin protein in response to DHI. The thermal stability of vinculin protein was not affected by DHI in the various temperatures and concentrations tested. As a positive control, we measured the thermal stability of IKK*β* in response to ainsliadimer A, a reported IKK*α/β* inhibitor (Supplementary Figure S6). We observed that the thermal stability of IKKβ could be reduced in this condition. Together, these results suggest that DHI directly interacts with IKK*α/β* in NHL cells.

**Figure 6:**
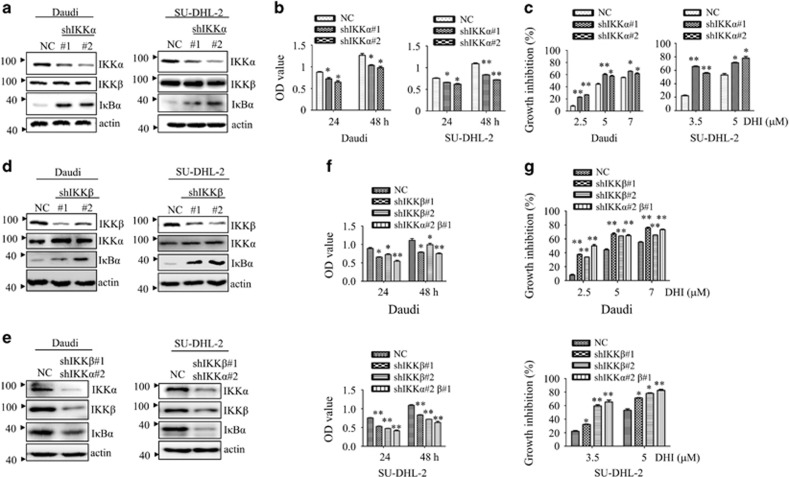
IKK*α/β* knockdown enhances the effect of DHI in NHL cells. (**a**, **d** and **e**). Daudi and SU-DHL-2 cells were stably infected with NC, shIKK*α* (**a**), shIKK*β* (**d**) or shIKK*α/β* (**e**). After transfection, the indicated proteins were tested by western blotting. *β*-actin was used as loading control. (**b** and **f**). Equal amounts of these stably infected lymphoma cells were cultured in 96-well plates. After 24 and 48 h, cell proliferation was analyzed by CCK-8. (**c** and **g**). The effect of DHI on cell growth in NC-, shIKK*α*- (**c**) and shIKK*β*- (**g**) transfected Daudi and SU-DHL-2 cells were determined by CCK-8 after 24 h of treatment. All values represent the means±S.D. of three independent experiments. *P<0.05; **P<0.01 *versus* the control

To investigate whether targeting IKK*α/β* contributes to DHI’s ability to inhibit cell proliferation, IKK*α/β* was knocked down in lymphoma cells. As shown in Figures 6a, d and e, knockdown of IKK*α/β* in Daudi and SU-DHL-2 cells specifically reduced the protein level of IKK*α/β*. Interestingly, silencing only IKK*α* or IKK*β* increased the protein level of I*κ*B*α* and decreased p-I*κ*B*α*, whereas silencing both of them decreased I*κ*B*α* (Supplementary Figures S7a and b). Though the underlying mechanism of decreased I*κ*B*α* in both IKK*α*/IKK*β* silenced cells is currently not known, one possibility is that by reducing the expression of both IKK*α* and IKK*β*, the protein level of IκB*α* is regulated in an IKK*α/β* kinase-independent manner. Compared with the vector-transfected cells, knockdown of either IKK*α* or IKK*β* alone significantly inhibited cell growth (Figures 6b and f.) and enhanced DHI-induced inhibition of cell proliferation (Figures 6c and g). This effect could be further enhanced by knockdown of both IKK*α* and IKK*β* (Figures 6f and g).

### Overexpression of p65, p52 or RelB partially abrogates the anti-proliferation effect of DHI on NHL cells

**Figure 7:**
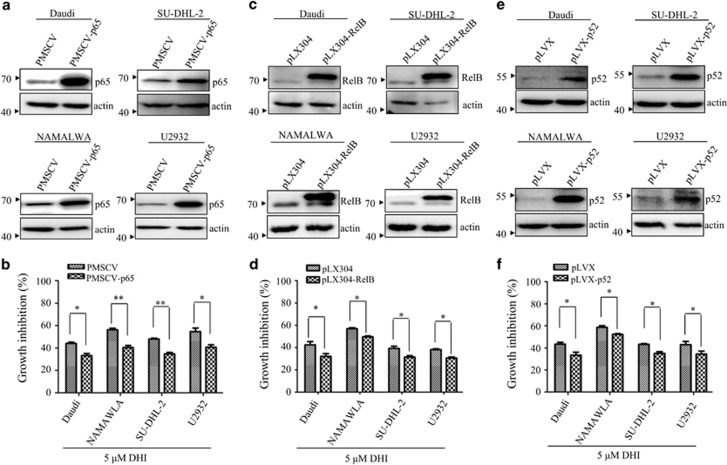
Overexpression of components of NF-κB complex partially abrogate DHI-induced NHL cells proliferation inhibition. (**a**, **c** and **e**) Ectopic p65, p52 or RelB and its corresponding vectors were stably expressed in Daudi, NAMALWA, U2932 and SU-DHL-2 cells. The indicated proteins were detected by western blotting. (**b**, **d** and **f**) These cells were treated with DHI for 24 h and cell proliferation was analyzed by CCK-8. All values represent the means±S.D. of three independent experiments. *P<0.05; **P<0.01 *versus* the control

Both the classical and alternative NFκB pathway could be regulated by IKK*α/β*. P65, p50, RelB are the important downstream substrates of IKK*α/β*. Intriguingly, knockdown of IKK*β* reduced the protein level of phosphorylated p65 in cytoplasm but did not alter the amount of nuclear p-p65 protein in NHL cells (Supplementary Figure S8a), however, it reduced nuclear p65 (Supplementary Figure S8b). To determine whether the reduction of nuclear p65 is associated with proliferation inhibitory effect of DHI on NHL cells, p65 was overexpressed in NHL cell lines. As shown in Figures 7a and b, overexpression of p65 partially abrogated the proliferation inhibitory effect of DHI. Furthermore, overexpression of NF-κB p52 or RelB in NHL cells also can reduce the inhibitory effect of DHI on the NHL cells (Figures 7c, d, e and f), indicating blockade of classical and alternative NF-κB pathway is involved in DHI-mediated growth inhibition of NHL cell lines.

### DHI inhibits lymphoma tumor growth in xenograft mice models.

**Figure 8:**
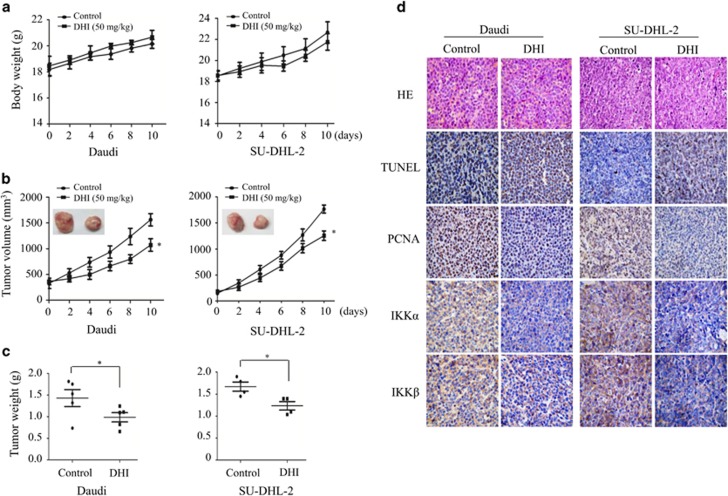
DHI inhibits NHL tumor growth in xenograft mice model. B-NSG mice were inoculated subcutaneously with Daudi or SU-DHL-2 cells. When solid tumors grew to ~100 mm^3^, the mice received intraperitoneal injections of either vehicle control or DHI (50 mg/kg) daily for 10 days. The mice weights (**a**), tumor volume (**b**) and tumor weights (**c**) were measured. The symbols * and ** indicate *P*<0.05 and 0.01, respectively, compared with the vehicle injected mice. (**d**) Expression patterns of PCNA, TUNEL and IKK*α/β* were examined by immunohistochemistry in the xenograft tumors on day 10 in each group. Original magnification, × 1000

To further explore the effect of DHI *in vivo*, xenograft mice models were established by the subcutaneous injection of Daudi and SU-DHL-2 cells in B-NSG mice which are NOD-SCID IL-2 receptor gamma null mice. When tumors grew to 100 mm^3^, mice were injected intraperitoneally with DHI (50 mg/kg) or vehicle daily for 10 days. Both tumor volume and mouse body weight were measured every other day. DHI significantly inhibited tumor growth (Figure 8b). At the end of treatment, tumor weight of the DHI-treated group was significantly lower than that of the vehicle-treated group (Figure 8c) and just a slight body weight loss was observed in DHI-treated mice (Figure 8a). IHC analysis showed that protein levels of IKK*α/β* and the proliferating nuclear cell antigen (PCNA), a marker of proliferation, were significantly decreased in the DHI-treated tumor (Figure 8d). We also observed an increase of TUNEL-positive cells in DHI-treated tumors compared to those from the vehicle condition, which indicates the induction of apoptosis (Figure 8d). These results suggest that DHI has anti-tumor activity in NHL xenograft models.

## Discussion

Aberrant NF-*κ*B activation underlies the development of many cancers^30^ and has been associated with tumor cell survival, proliferation, invasion and angiogenesis,^31^ especially in lymphoid malignancies. Targeting NF-*κ*B signaling pathways represents a promising strategy in the treatment of NHL.^14^ In the present study, we demonstrated that DHI, a small natural compound, induces proliferation inhibition and apoptosis of NHL cells *in vitro* and *in vivo*. Moreover, we provide evidence that the anti-NHL effect of DHI is associated with inhibition of IKK*α/β* and other survival pathways.

The IKK complex is composed of IKKα and IKKβ catalytic subunits and a regulatory subunit, IKKγ/NEMO. Its key function is to phosphorylate I*κ*Bs and the NF-*κ*B precursors, p105 and p100, which then leads to the activation of NF-*κ*B.^25^ In recent years, an IKK kinase-independent function was also found.^32, 33^ As this complex has a crucial role in cancer, as well as inflammatory and autoimmune disease, significant efforts have been made to identify IKK inhibitors.^25, 34^ Many compounds have now been introduced as IKK inhibitors, such as celastrol, PS1145, BMS345541 and others.^35, 36, 37, 38, 39, 40^ Inhibition of IKK can inhibit the proliferation of cancers or sensitize cancer cells to chemotherapeutic drugs.^41, 42^ In this study, we demonstrated that IKK*α* and IKK*β* are direct targets of DHI, which was supported with the following evidence: CETSA results unambiguously showed that DHI interacts with IKKα and IKK*β*. CETSA, a recently developed method to assess drug engagement in complex environments,^28, 29^ builds on the discovery that ligand induced protein thermal shift can also be measured in the context of cell lysates.^43, 44, 45^ Compared with *in vitro* assays using recombinant proteins, CETSA keeps target proteins in their native, local environments, maintaining their original post-translational modifications and expression level. Using this method, we found that DHI reduces the thermal stability of IKK*α/β*, indicating that DHI can interact with IKK*α/β* in cells. Consistent with this result, DHI treatment inhibited the phosphorylation and degradation of I*κ*Bα and also inhibited the phosphorylation of NF-*κ*B, which in turn inhibited the activation of NF-*κ*B and thus the related, downstream functions of NF-*κ*B. In addition to the classical pathway, IKK*α* plays an important role in regulating the activation of the alternative NF-*κ*B pathway, it is possible that DHI can also inhibit the alternative pathway. In support of this opinion, we found that both total and nuclear p52, the critical component of alternative pathway, were significantly downregulated by the treatment of DHI (Supplementary Figures S10a and b). In addition, similar to the inhibition induced by DHI, IKK*α/β* knockdown suppress NHL cells proliferation. Unexpectedly, IKK*β* knockdown decreases p65 in nucleus, but did not change the nuclear p-p65 and overexpression of p65 partially abrogates the proliferation inhibitory effect of DHI on NHL cell lines, these data suggest that the inhibitory effect of knockdown of IKK*α/β* might be p65 phosphorylation independent. Similar observations were reported that the phosphorylation of serine 536 was not required for p65-mediated protection from TNFα cytotoxicity and Traf1 induction in fibroblasts.^46^ One interesting observation is that DHI treatment decreased the protein level of IKK*α/β*, which could not be reversed by proteasome inhibitor MG132 or chloroquine (Supplementary Figure 11), indicating that other ways beyond proteasome system or lysosomal system participates in this process. The underlying mechanism need to be further explored. Compared with the other inhibitors that only impede kinase activity, DHI may not only block IKK kinase activity but also abrogate the kinase-independent activity of IKK.^32, 33^ Moreover, it has been shown that mutation of IKK can confer cancer cell resistance to an IKK inhibitor.^47^ Therefore, DHI may provide an alternative approach to overcome this drug resistance. Most importantly, we demonstrate that DHI exhibits a significant anti-tumor effect *in vivo* with only mild liver toxicity (Supplementary Figure S9). Thus, targeting IKK*α/β* and thereby inhibiting the activation of NF-*κ*B may contribute to the tumoricidal properties of DHI. DHI has not been previously reported to have any anti-tumor effect, our results indicate that DHI is a novel potent compound in the treatment of NHL.

Another interesting finding in this work is that NAMALWA and SU-DHL-2 cells are more sensitive to DHI treatment than Daudi and SU-DHL-4 cells. We assumed that the degree of sensitivity might be associated with differing NF-*κ*B signaling components in each cell. Notably, the expression pattern of NF-*κ*B signaling components is similar in NAMALWA and SU-DHL-2 cells (Supplementary Figure S12). In the BL cell lines, IKK*α/β*, p65 and p50 were more highly expressed in NAMALWA cells than in Daudi cells, whereas I*κ*B*α* exhibited lower expression in NAMALWA cells (Supplementary Figure S12a), which suggests that the level of NF-*κ*B activation was higher in NAMALWA cells. In the DLBCL cell lines, the expression of NF-*κ*B components (IKK*α*, IKK*β*, p65, p-p65, p50 and p-I*κ*B*α*) was more highly upregulated in SU-DHL-2 cells than in SU-DHL-4 cells (Supplementary Figure S12b), indicating that NF-*κ*B was constitutively activated in SU-DHL-2 cells.^24^ Overall, these results support the idea that the different sensitivity of NHL cells to DHI treatment depends on their respective NF-*κ*B activity. It has been previously shown that SU-DHL-2 (ABC-DLBCL) cells are more resistant to R-CHOP treatment than are SU-DHL-4 (GCB-DLBCL) cells.^48^ Our results suggest that DHI might be used to overcome the R-CHOP resistance that arises in ABC-DLBCL cells. Besides the NF-*κ*B, BCR signaling pathways also have an important role in the survival of lymphoma cells. As overexpression of components of NF-*κ*B only partially abrogate the effect of DHI, we have also tested whether the BCR signaling pathway are also involved. It was found that DHI could evoke or repress the activity of AKT or ERK in a cell type dependent manner, meanwhile, the CETSA indicates that DHI does not directly interact with AKT and ERK protein (Supplementary Figure S4b). Further, we found that combined treatment with either AKT inhibitor MK2206 or ERK inhibitor U0126 enhance the inhibitory effect of DHI on SU-DHL-2 and Daudi cells (Supplementary Figures S4c and d). Taken together, we propose that the anti-NHL effect of DHI may be a combined effect from its influence on the NF-*κ*B signaling pathway, PI3K/AKT and ERK pathways or other unknown signaling pathways.

In summary, our findings highlight the potential clinical benefit of DHI as a chemotherapeutic agent against NHL, particularly for those patients with IKK-related aberrant NF-*κ*B activation.

## Materials and methods

### Cell lines and reagents

The human BL cells – Daudi and NAMALWA – and the DLBCL cells – SU-DHL-4 (GCB-DLBCL), SU-DHL-2 (ABC-DLBCL), OCI-Ly8 (GCB-DLBCL), and U2932 (ABC-DLBCL) – as well as HeLa cells were purchased from the American Type Culture Collection (ATCC, Manassas, VA, USA). Cells were cultured in either RPMI 1640 (NHL cells) or Dulbecco-modified minimum essential medium (DMEM) (HeLa cells) supplemented with 10% (w/v) fetal bovine serum (FBS; Gibco, Grand Island, NY, USA) and 1% penicillin–streptomycin (Gibco) at 37 °C in a humidified atmosphere with 5% CO_2_. DHI, MK2206 (Selleck Chemicals, Houston, TX, USA) and U0126 (Selleck Chemicals) were dissolved in DMSO.

### Cell viability assay

The effect of DHI on cell viability was determined by trypan blue exclusion assay. Trypan blue exclusion assay measuring cell viability is based on the principle that live cells with intact membranes are able to exclude the dye, whereas dead cells without an intact membrane accept the dye. NHL cells (5 × 10^5^/ml per well) were seeded in 6-well plates and culture medium containing vehicle or DHI was added to each well. Cells were incubated at 37 °C. At the indicated time points (0, 24, 48 h), cells were suspended with 0.05 % trypsin-EDTA and stained with trypan blue dye (0.4%). The unstained (viable) and stained (dead) cells were counted in the Neubauer hemocytometer. The cell viability (%) was calculated as a ratio, the number of live cells relative to the total number of cells. Experiments were repeated three independent times, and the data were presented as bar diagram with standard deviation.

### Cell proliferation assay

Cell proliferation activity was determined by CCK-8 assays, according to the manufacturer’s instructions. NHL cells were plated at a certain density in 96-well plates with either the proper vehicle control or with different concentrations of DHI for 24 h. Each condition was run in three duplications. Next, 10 *μ*l of CCK-8 solution (CK04, Dojindo Molecular Technologies, Kumamoto, Japan) were added to the wells, and the plates were incubated for 4–6 h. Absorbance (OD) was read at 450 nm using an Elx 9808 microplate reader (Bio-Tek, Winooski, VT, USA). The ratio of growth inhibition was calculated by the following formula: control OD value minus the OD value of the drug treatment divided by the control OD value.

### Cell cycle assay

Propidium iodide (PI, Sigma, Louis, MO, USA) staining was used to analyze cell cycle distribution. After exposure to different concentrations of DHI for 24 h, cells were harvested and fixed with 70% ethanol at −20 °C overnight. Cells were then washed twice with phosphate-buffered saline (PBS), incubated with PBS containing 10 *μ*l RNase A (25 μg/ml) at 37 °C for 30 min, and stained with PI (1 mg/ml in PBS). The DNA content of cells and cell cycle distribution were analyzed by Flow cytometric analysis on BD LSRFortessa cell analyzer (BD Biosciences, San Diego, CA, USA).

### Cell apoptosis assay

The apoptosis rate of NHL cells was determined using Annexin V-FITC and PI (BD Pharmingen, San Jose, CA, USA) staining. NHL cells (5 × 10^5^/ml) in 6-well plates were treated with different concentrations of DHI for 24 h. Cells were collected, washed with PBS three times, and resuspended in 400 *μ*l of 1 × binding buffer. Next, 5 *μ*l of Annexin V-FITC and 5 *μ*l of PI were added to each sample, and the samples were incubated in the dark for 15 min. Cells were analyzed by fluorescence-activated cell sorting using a BD LSRFortessa cell analyser (BD Biosciences). Cells that stained positive for early apoptosis (Annexin V-FITC-stained only) and for late apoptosis (Annexin V-FITC-and PI-stained) were combined for analysis, and the results were analyzed using FlowJo 7.0 software (Tree Star, Ashland, OR, USA).

### ShRNA-mediated IKK*α*/ *β* knockdown

The shRNA or NC plasmids were cotransfected with the lentiviral packaging vectors PSPAX2 and pMD2G and introduced into HEK293T cells to produce lentivirus. After 48 h, the viral supernatant was collected and added to lymphoma cells in six-well plates with medium containing 8 *μ*g/ml polybrene (Sigma). The infected cells were spun at 1000 × *g* for 100 min, and fresh media was added. After 2 days’ infection, stably transfected cells were selected with puromycin. The targeting sequences of shIKK*α/β* were shown to be the following:

shIKK*α*#1, 5′-AAGCAGAAGATTATTGATCTA-3′

shIKK*α*#2, 5′-CAGGAGAAGTTCGGTTTAGTA-3′

shIKK*β*#1, 5′-CGCTTAGATACCTTCATGAAA-3′

shIKK*β*#2, 5′-CTGGATGA CATCTTAAACTTA -3′

### Plasmid p65, p52, RelB and overexpression

P65 gene was amplified from HEK293T cells by reverse transcription PCR and the PCR product was cloned into PMSCV vector to create p65 plasmid. P52 gene was amplified from the p100 plasmid (kindly gift from Dr. Xiaodong Yang, Shanghai Jiaotong University School of Medicine) and then cloned into Plvx-IRES-ZsGreen1 vector to form p52 plasmid. RelB were purchased from the DNA library of Shanghai Jiao Tong University School of Medicine. The p65 or vector plasmids were cotransfected with retrovirus packaging vectors Gag-Pol and VSVG and introduced into HEK293T cells to produce retrovirus. P52, RelB and its corresponding vector were cotransfected with lentiviral packaging vectors PSPAX2 and pMD2G and introduced into HEK293T cells to produce lentivirus. The infection of NHL cells was performed as described above.

### Luciferase assay

Daudi cells were stably infected with NF-*κ*B Luciferase reporter plasmid (Promega, Madison, WI, USA). Equal numbers of cells were plated in 24-well plates, then treated with various concentrations of DHI for 4 h, followed by a 4-h stimulation with or without TNF*α* (15 ng/ml). Cells were lysed in passive lysis buffer (Promega) for 15 min. The transcriptional activity was determined by measuring the activity of firefly luciferase in a multiwell plate luminometer (Tecan, Durham, NC, USA) using a luciferase reporter assay system (Promega) according to the manufacturers’ instructions.

### Real time quantitative RT-PCR

Total RNA was isolated using a TRIzol kit (Invitrogen, Carlsbad, CA, USA), and RNA was treated with DNase (Promega). cDNA was reverse transcribed using MMLV reverse transcriptase (Promega). Real time quantitative PCRs were performed with SYBR Green PCR Master Mixture reagents (Roche, Basel, Switzerland). The primer sequences are shown in Supplementary Table S1.

### Western blotting

The whole cell lysates were extracted in 1 × SDS, equally loaded to 8–12% SDS–polyacrylamide gel, electrophoresed, transferred to nitrocellulose membrane (Bio-Rad, Hercules, CA, USA) and then stained with 0.4% Ponceau S red to ensure equal protein loading. After blocking with 5% nonfat milk in PBS, the membranes were incubated with antibodies against IKK*α*, IKK*β*, p-p65, p105/p50, I*κ*B*α*, p-I*κ*B*α*, p-AKT(S473), ERK, p-ERK(Thr202/Tyr204), Caspase-3, Cleaved-Caspase-3 and *β*-actin (Cell Signaling Technology, Beverly, MA, USA), NF-*κ*B2, RelB (Proteintech, Rosemont, IL, USA), c-Myc, cyclin D1 (Abcam, Cambridge, UK), PARP, AKT, p65, cyclin A, vinculin, Lamin B (Santa Cruz Biotech, Santa Cruz, CA, USA), overnight at 4 °C, followed by HRP-linked secondary antibody (Millipore, Billerica, MA, USA) for 1 h at room temperature. The signals were detected with a chemiluminescence phototope-HRP kit (Cell Signaling Technology), used according to manufacturer’s instructions.

### Immunofluorescence analysis

The treated cells were fixed with 4% paraformaldehyde, treated with 0.3% Triton X-100, and blocked with 2% bovine serum albumin. Cells were then sequentially incubated with antibody NF-κB p65 (Santa Cruz Biotech, sc-109) or NF-κB1 (CST, 12540s) overnight at 4 °C, followed by FITC labeled anti-rabbit immunoglobulin G antibody (Invitrogen) and 4’,6-diamidino-2-phenylindole (Vector Laboratories, Burlingame, CA, USA). Stained cells were examined with immunoflurescence microscopy (Nikon, Tokyo, Japan).

### CETSA

Daudi cells or SU-DHL-2 cells were harvested and diluted in PBS supplemented with protease inhibitor cocktail (Calbiochem, Billerica, MA, USA). The cell suspensions were freeze–thawed three times in liquid nitrogen. The soluble fraction (lysates) was separated from the cell debris by centrifugation at 20 000 × *g* for 20 min at 4 °C. The cell lysates were divided into two aliquots. One aliquot was treated with DMSO and the other aliquot with DHI (100 *μ*M) or ainsliadimer A (10 *μ*M). After a 30 min incubation at room temperature, the respective lysates were divided into separate aliquots (30 μl) and heated individually at different temperatures for 3 min (Veriti thermal cycler, Applied Biosystems/Life Technologies, Foster City, CA, USA). This was followed by cooling for 3 min at room temperature. The appropriate temperatures were determined in preliminary CETSA experiments (data not shown). The heated lysates were centrifuged at 20 000 × *g* for 30 min at 4 °C in order to separate the soluble fractions from the precipitates. The supernatants were transferred to fresh microtubes and analyzed by sodium dodecyl sulfate polyacrylamide gel electrophoresis (SDS-PAGE) followed by western blotting analysis. Dose effect of DHI on the thermal stability of IKK*α/β* was evaluated as follows. After the cell lysates solution was obtained via the above method, we divided the lysates solution into six uniform parts and then added equal volumes of the corresponding concentration of DHI, followed by 30 min incubation. Subsequently, we heated the lysates solution incubated with various concentrations of DHI at 54 °C for 3 min (Veriti thermal cycler, Applied Biosystems/Life Technologies), followed by cooling for 3 min at room temperature. The heated lysates were centrifuged at 20 000 × *g* for 30 min at 4 °C to separate the soluble fractions from the precipitates. The supernatants were transferred to new microtubes and analyzed by SDS-PAGE followed by western blotting analysis.

### Animal experiments

B-NSG mice, NOD-SCID IL-2 receptor gamma null mice, 6–8 weeks old were obtained from Jiangsu Biocytogen Co., Ltd (Nantong, China). Daudi (1 × 10^7^) cells and SU-DHL-2 (2 × 10^7^) cells were injected into the right flank of the B-NSG mice, respectively. After 5–10 days of inoculation, mice were randomly divided into two groups. One group was dosed intraperitoneally with DHI (50 mg/kg) and the other was injected with vehicle (administrated daily). The treatment period lasted for 10 days. Tumor volumes and mice body weights were monitored every two days. At the end of DHI treatment, mice were killed, and tumors were excised and weighed. Tumor volume was calculated by using the formula mm^3^=1/2 *a* × *b*^2^, where *a* is the length and *b* is the width. All animals were handled according to the protocols approved by the Committee for the Humane Treatment of Animals at Shanghai Jiao Tong University School of Medicine.

### Immunohistochemical analysis

The tumors of the mice treated with vehicle or DHI were formalin fixed, paraffin embedded and sectioned into slices. Tissue sections were stained with hematoxylin and eosin, and then the immunohistochemistry for IKK*α/β*, PCNA and TUNEL were performed on the sections.

### Statistical analysis

All graph preparations and statistical calculations were performed using GraphPad Prism 5.0 software (GraphPad Software Inc., La Jolla, CA, USA). Student’s *t*-test was used to evaluate differences between two groups. *P*-value<0.05 was regarded as statistically significant.
