# Supplementary figures and images for: Identification of 11(13)-dehydroivaxillin as a potent therapeutic agent against non-Hodgkin's lymphoma
Source: Cell Death Dis. 2017 Sep 14;8(9):e3050–. doi: 10.1038/cddis.2017.442 (PMC5636986; doi:10.1038/cddis.2017.442)

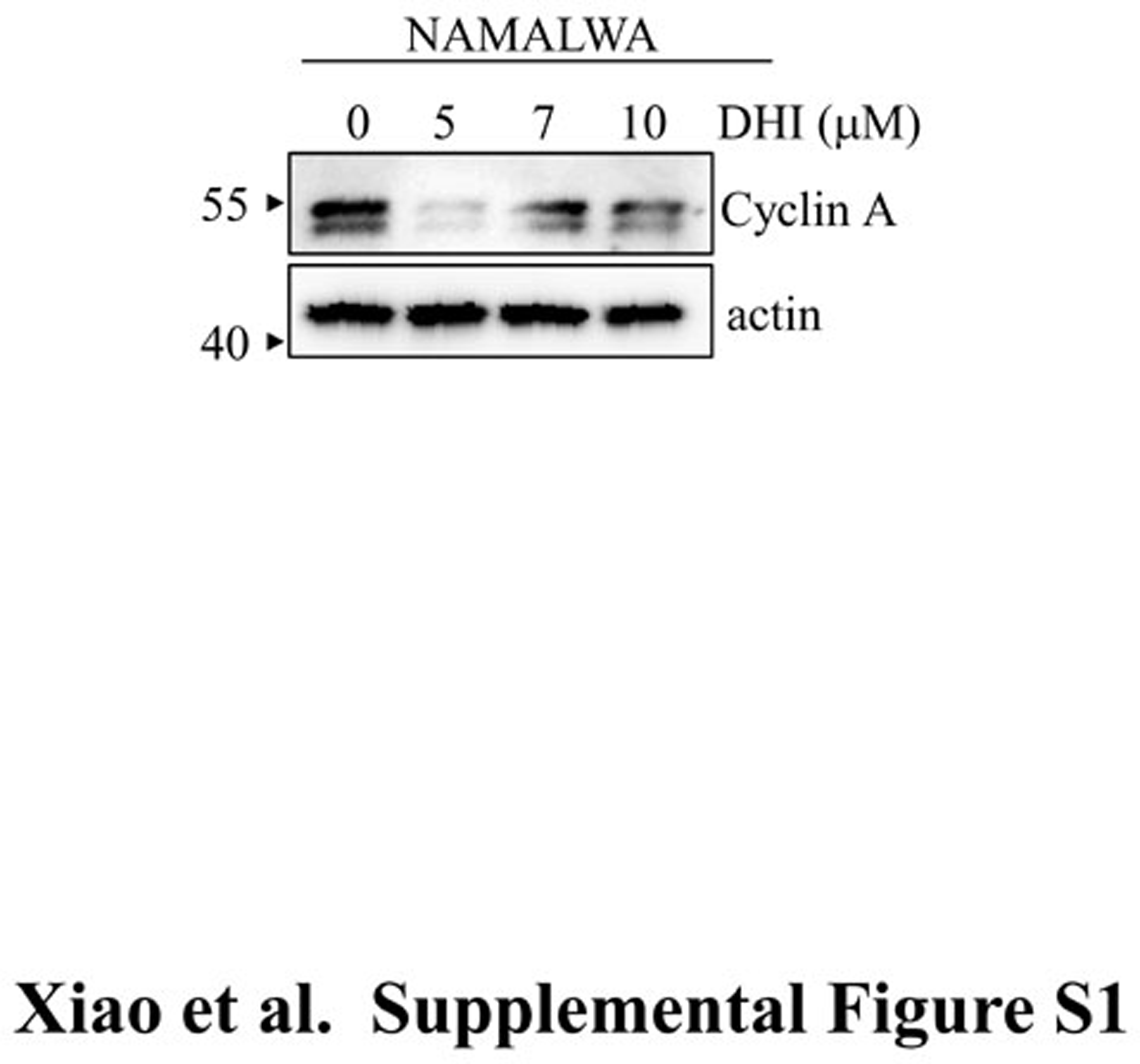

Supplement: Supplementary Figure S1 [file cddis2017442x1.tif]

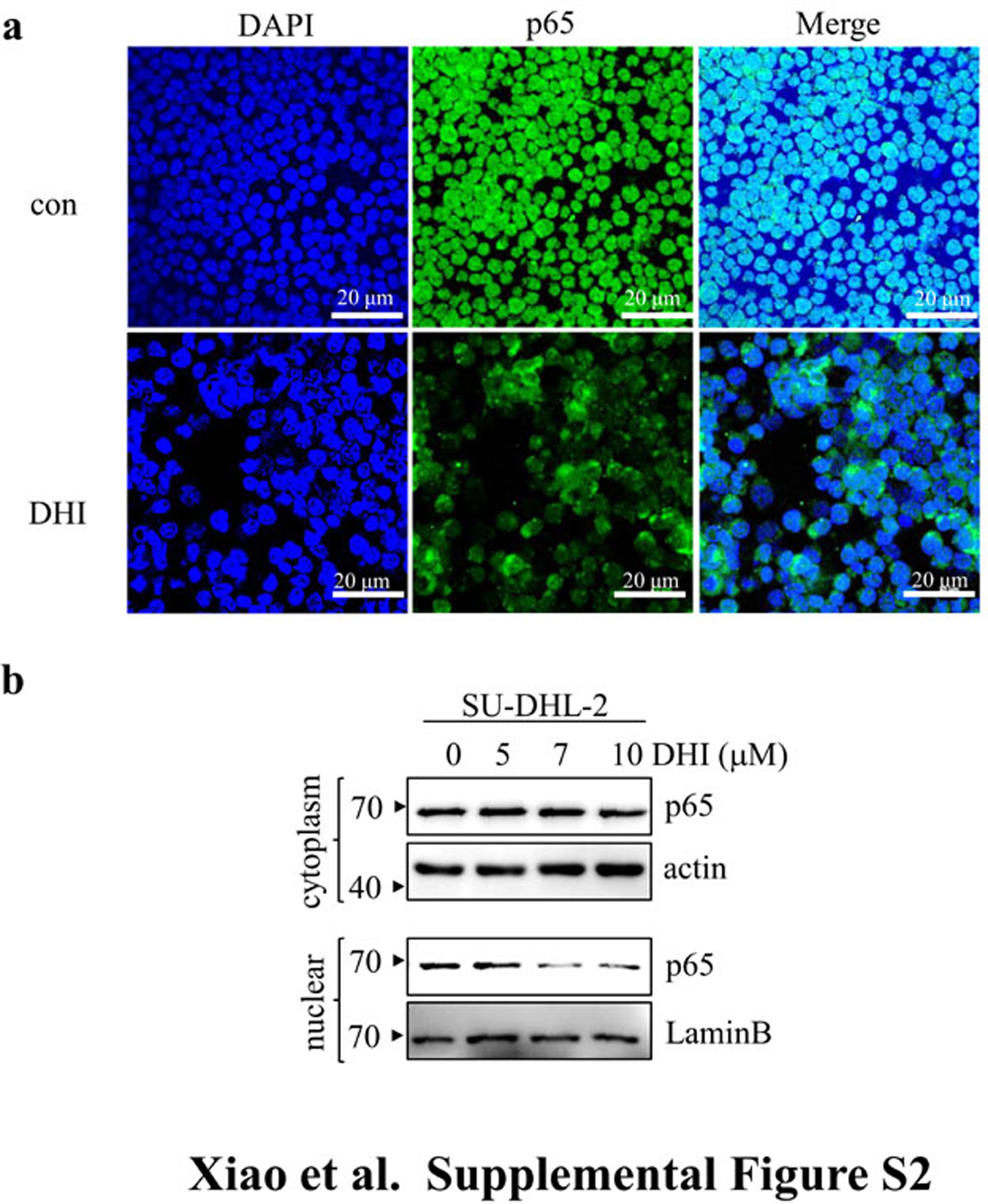

Supplement: Supplementary Figure S2 [file cddis2017442x2.tif]

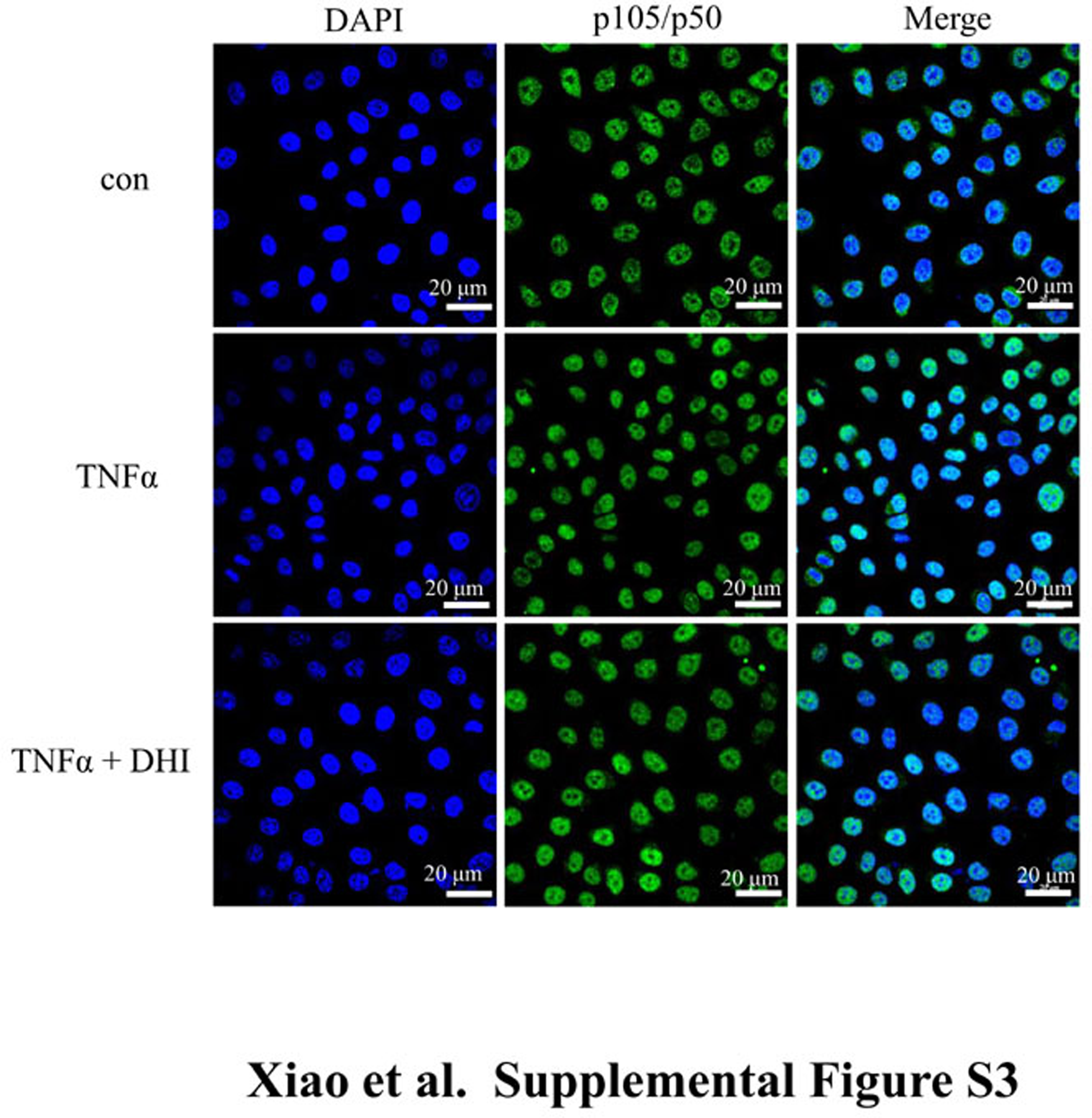

Supplement: Supplementary Figure S3 [file cddis2017442x3.tif]

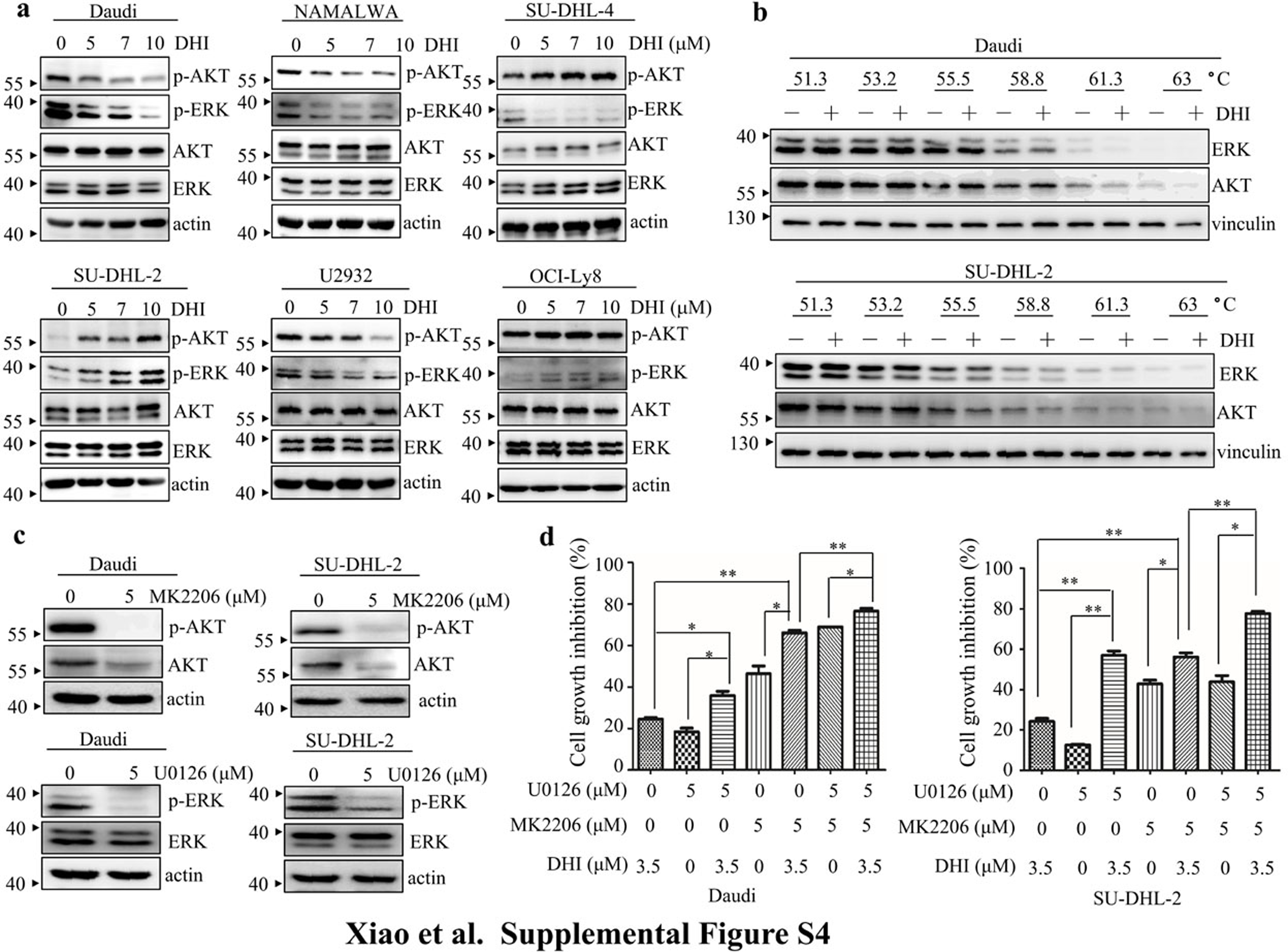

Supplement: Supplementary Figure S4 [file cddis2017442x4.tif]

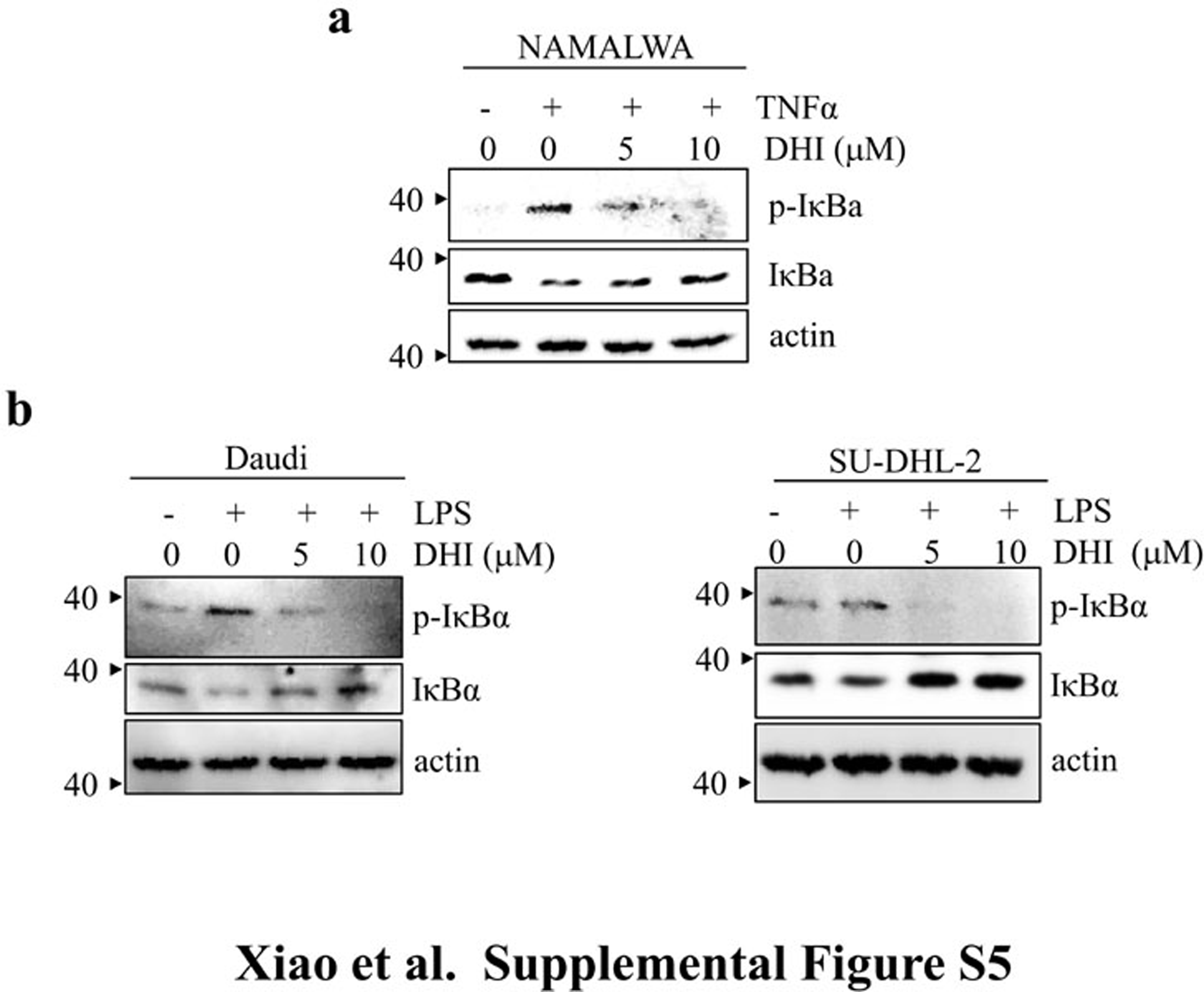

Supplement: Supplementary Figure S5 [file cddis2017442x5.tif]

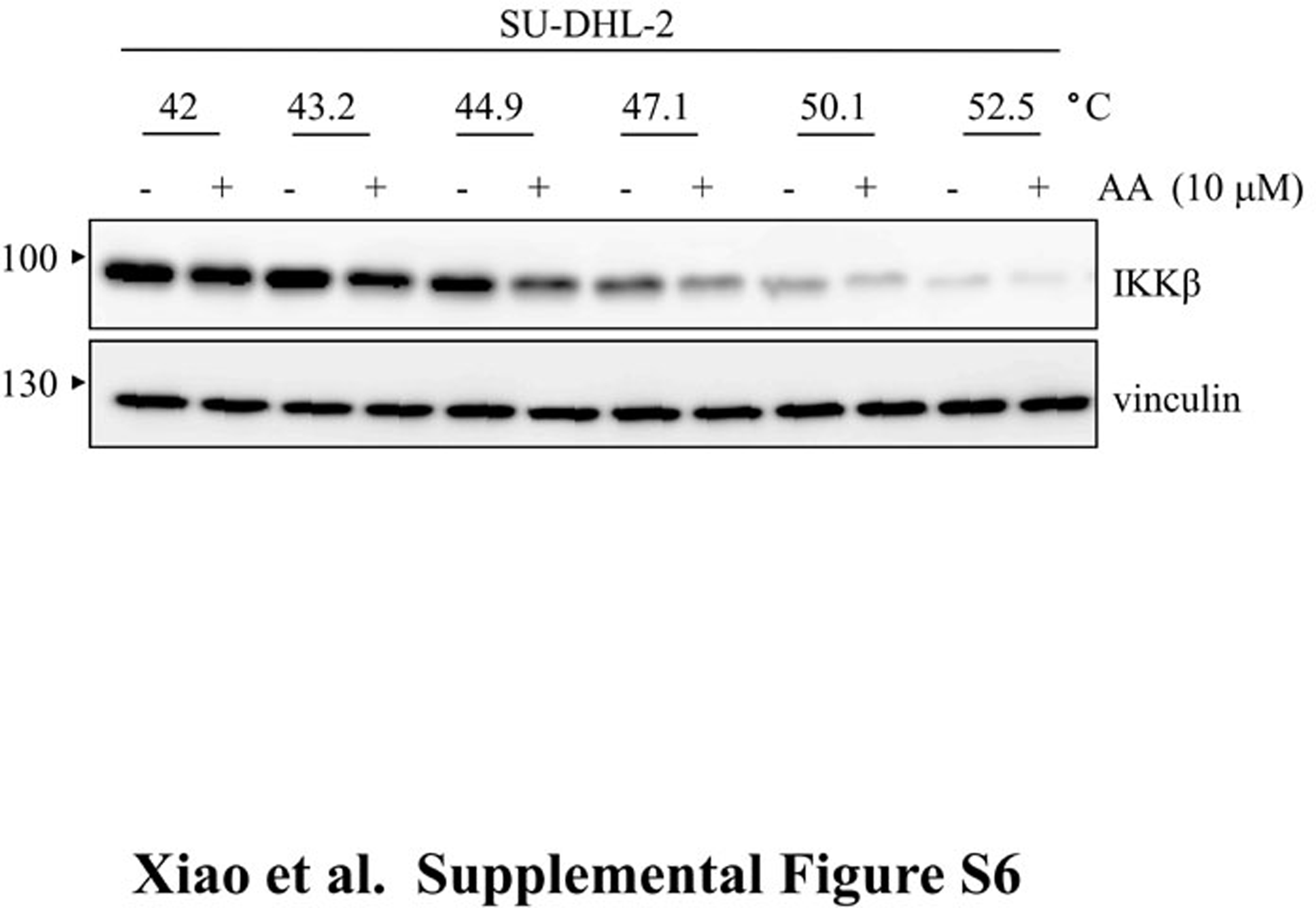

Supplement: Supplementary Figure S6 [file cddis2017442x6.tif]

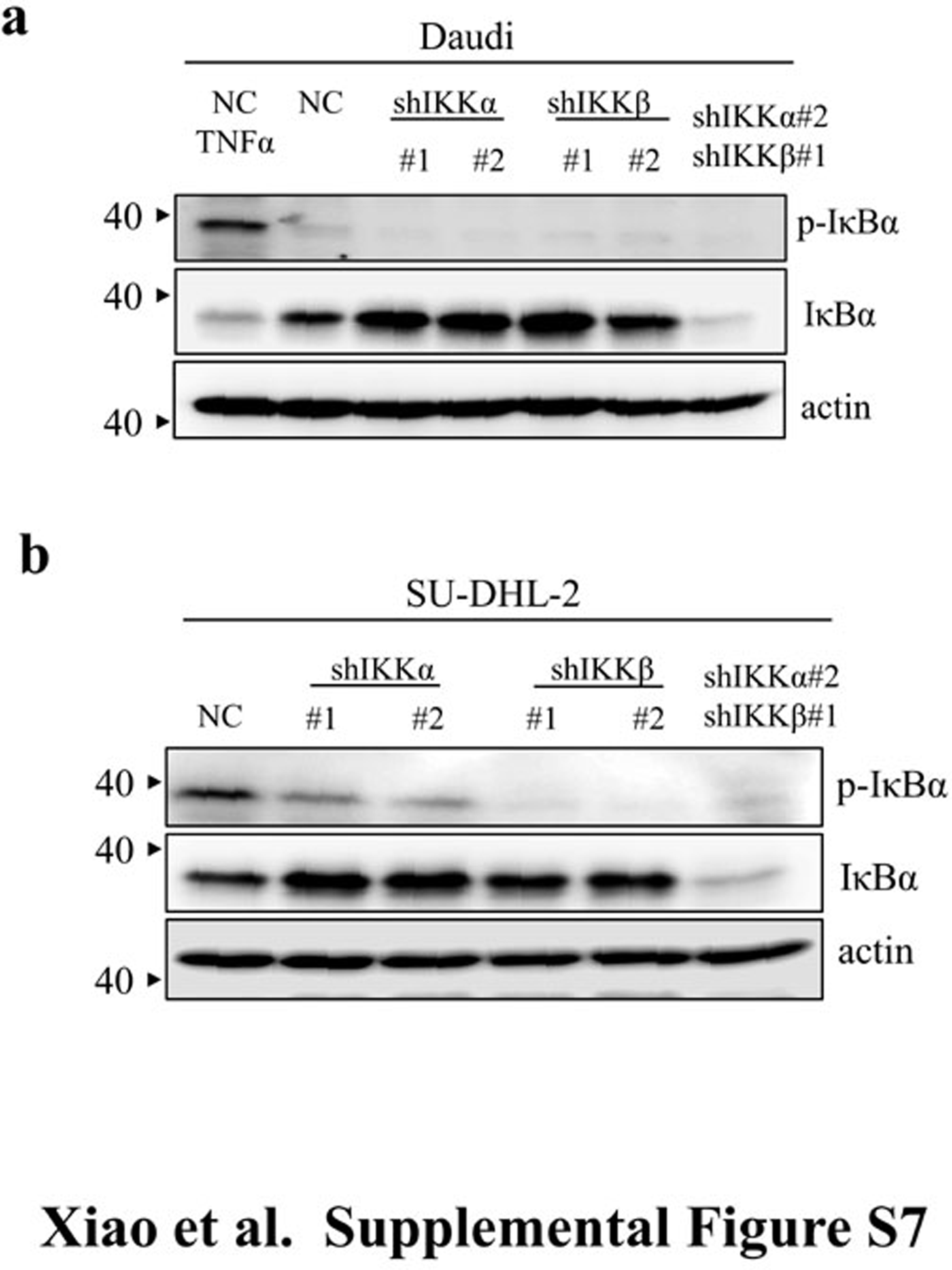

Supplement: Supplementary Figure S7 [file cddis2017442x7.tif]

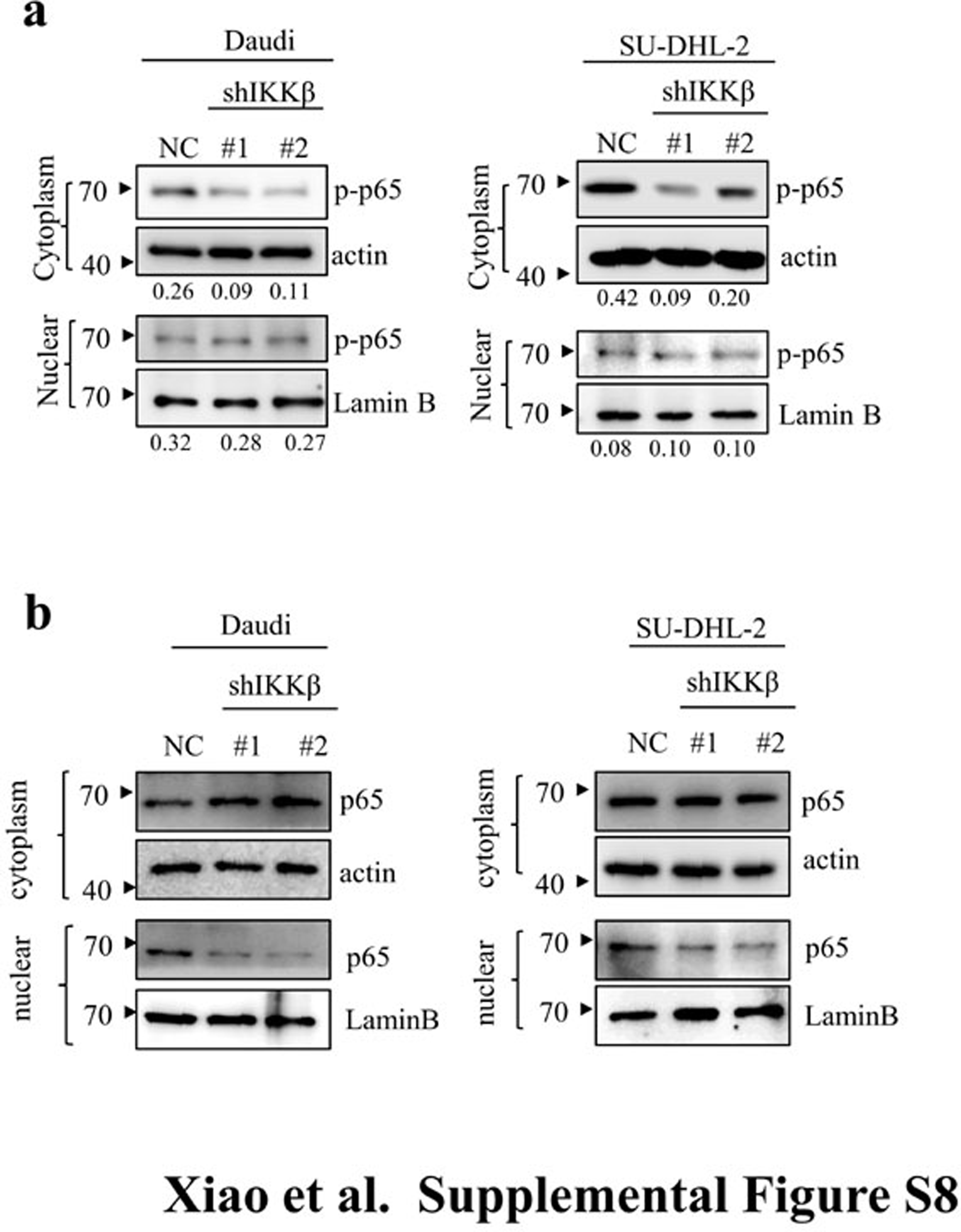

Supplement: Supplementary Figure S8 [file cddis2017442x8.tif]

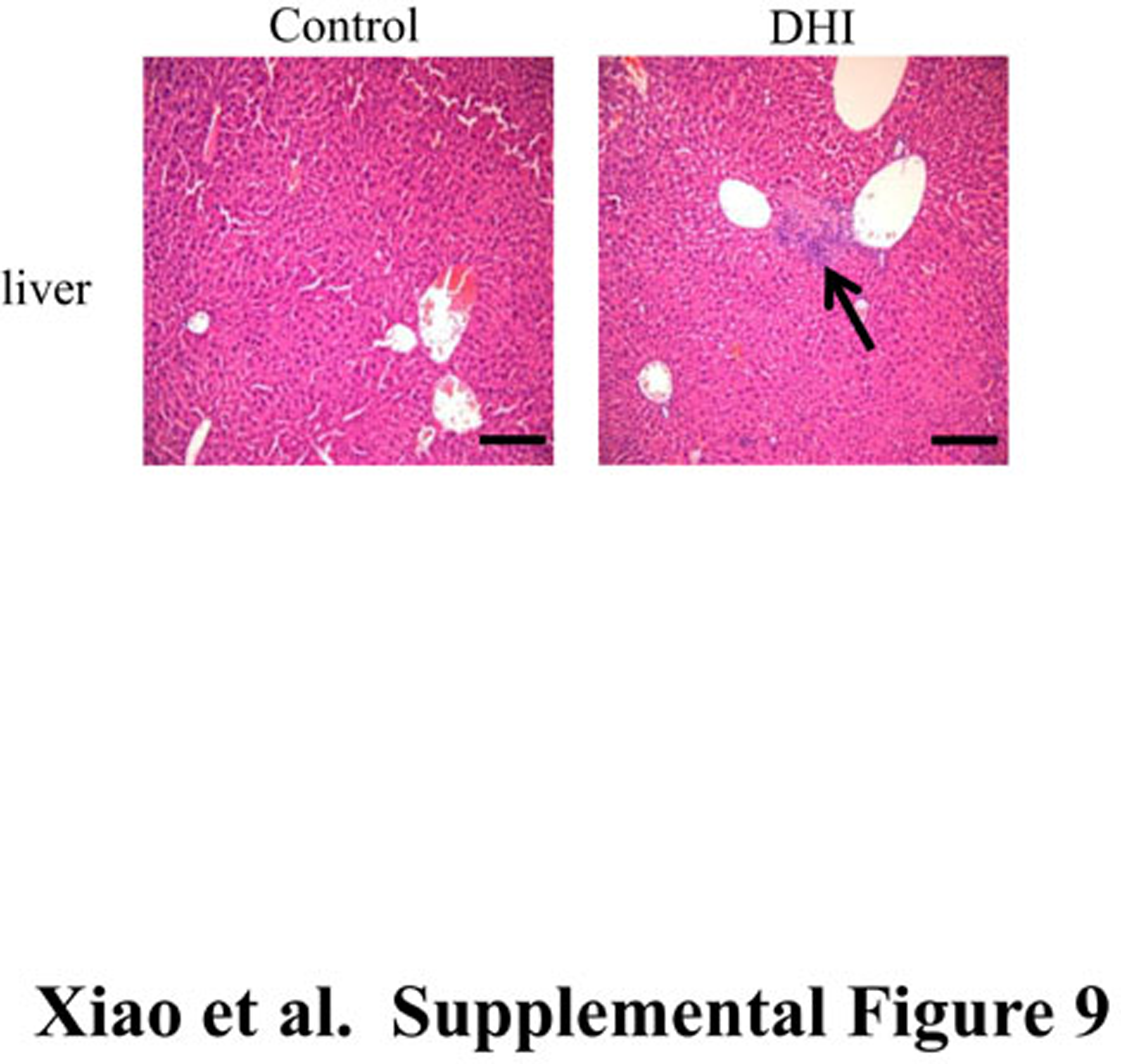

Supplement: Supplementary Figure S9 [file cddis2017442x9.tif]

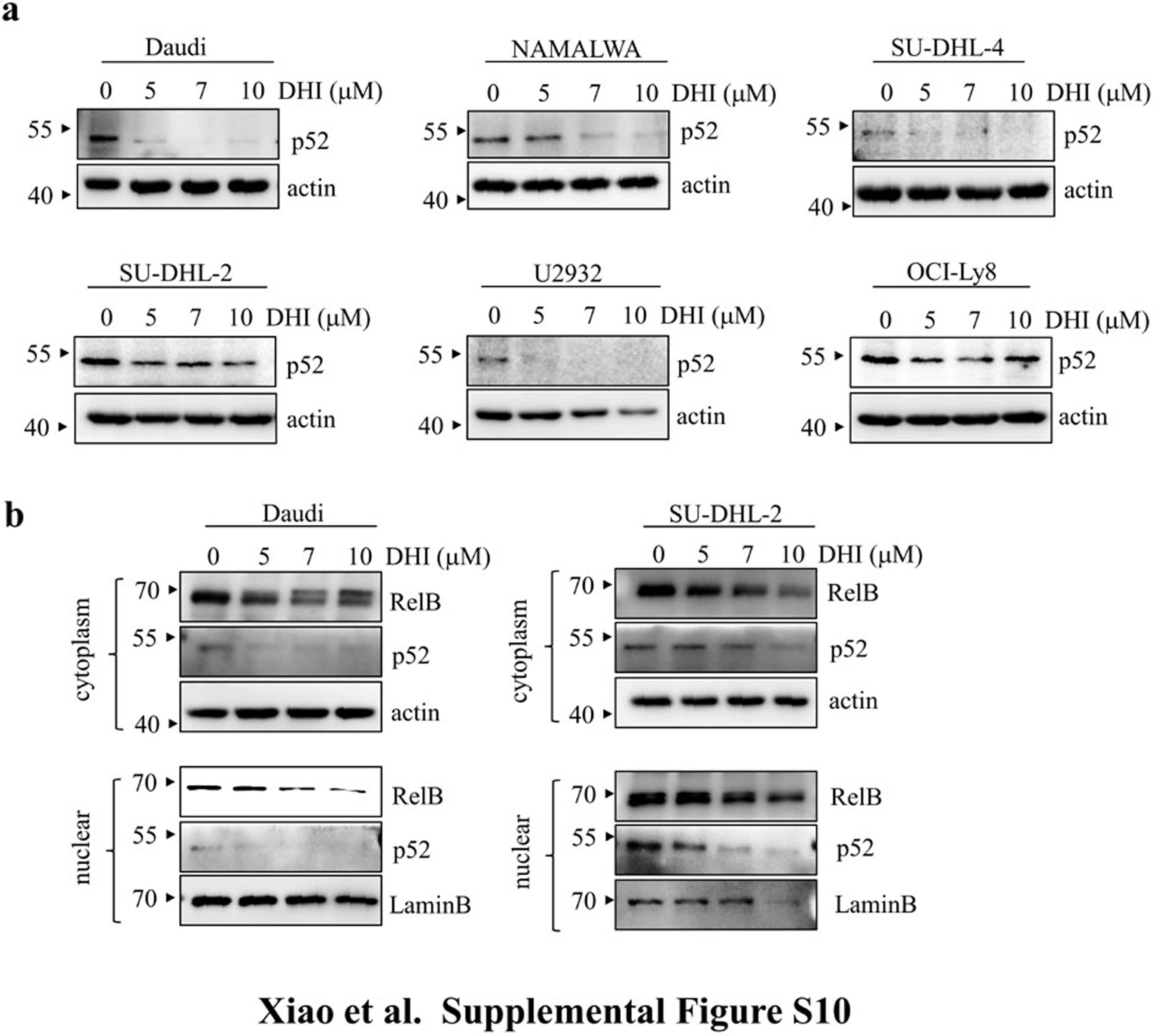

Supplement: Supplementary Figure S10 [file cddis2017442x10.tif]

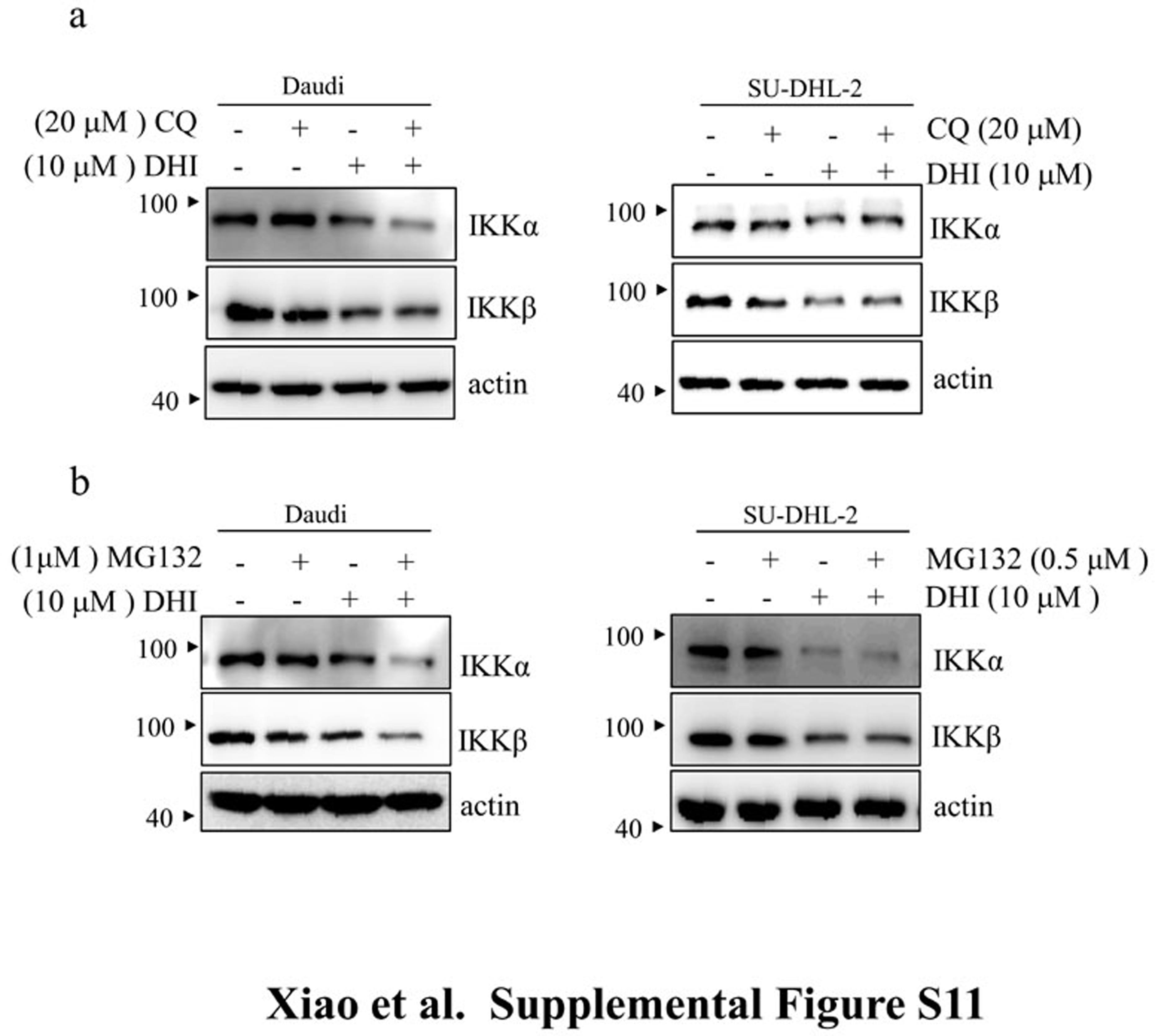

Supplement: Supplementary Figure S11 [file cddis2017442x11.tif]

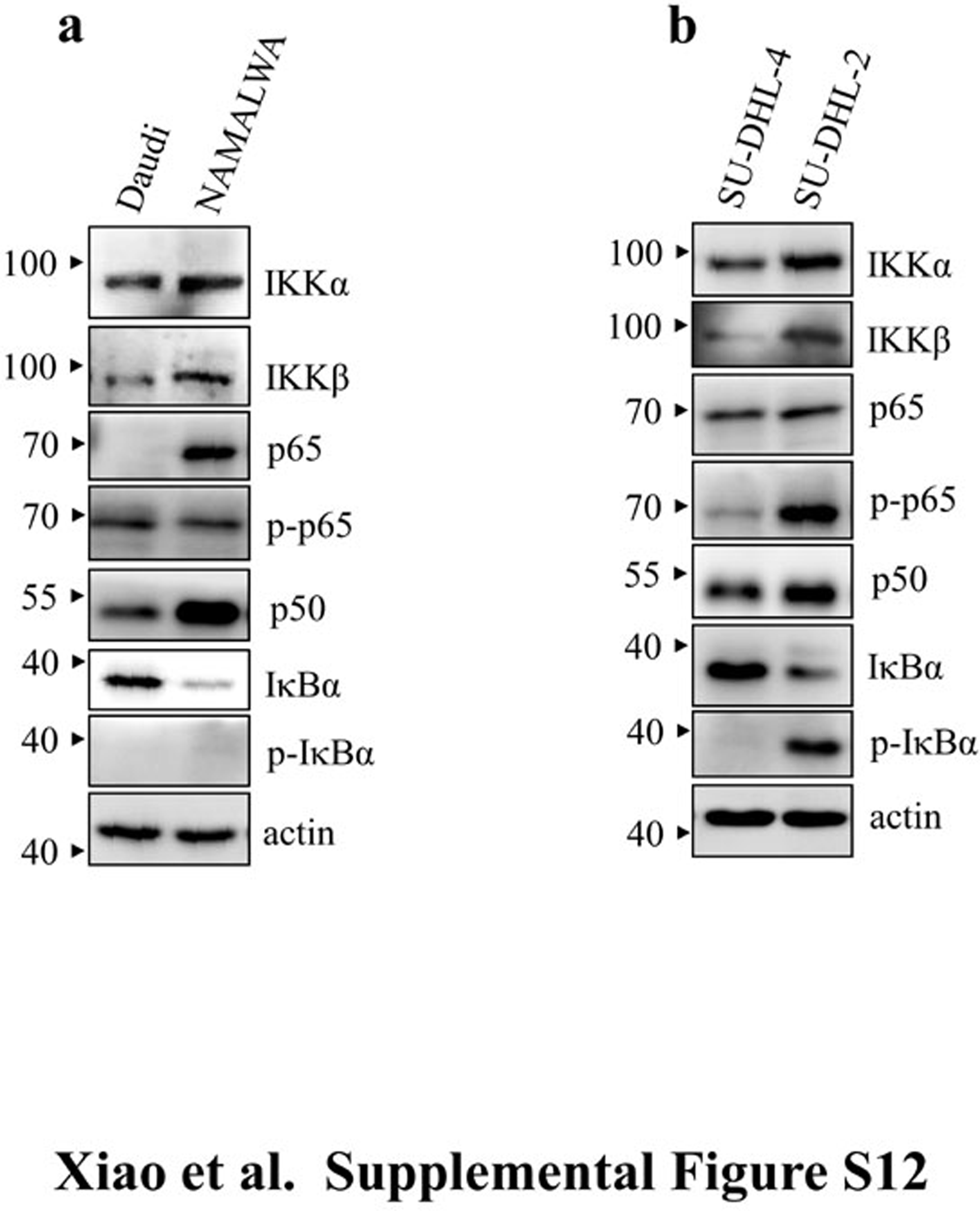

Supplement: Supplementary Figure S12 [file cddis2017442x12.tif]
