# Supplementary material for: Identification of 11(13)-dehydroivaxillin as a potent therapeutic agent against non-Hodgkin's lymphoma
Source: Cell Death Dis. 2017 Sep 14;8(9):e3050–. doi: 10.1038/cddis.2017.442 (PMC5636986; doi:10.1038/cddis.2017.442)
Supplement: Supplementary Material [file cddis2017442x13.pdf]

Identification of 11(13)-Dehydroivaxillin as a potent therapeutic agent against Non-Hodgkin's lymphoma

Xinhua Xiao[1, 2, 6], Huiliang Li[3, 6], Huizi Jin[5, 6], Jin Jin[2], Miao Yu[1], Chunmin Ma[2], Yin Tong[1], Li Zhou[4], Hu Lei[2], Hanzhang Xu[2], Weidong Zhang[3*], Wei Liu[1*] and Yingli Wu[2*]

Supplemental information

Supplemental Table S1

genes	Sense primers	Anti-sense primers
IκBα	CCTGGCCTTCCTCAACTTCC	CTGGCTGGTTGGGATCACAG
Bcl-2	CCGCTACCGCCGCGACTTC	AAACAGAGGCCGCATGGCTG
Cyclin D1	CCGCTGGCCATGAACTACCT	ACGAAGGTCTGCGCGTGTT
β-actin	CATCCTCACCCTGAAGTACCC	AGCCTGGATAGCAACGTACATG

Supplemental Figure S1. DHI decreases the expression of Cyclin A in NAMALWA cells. NAMALWA cells were treated with various concentrations of DHI for 24 hours and the indicated proteins were detected by western blotting. All experiments were repeated three times with the same results.

Supplemental Figure S2. DHI inhibits p65 nuclear translocation in NHL cells. (**a**). Immunofluorescent staining of p65 in SU-DHL-2 cells treated with or without DHI (10 μM) for 12 hours. (**b**). SU-DHL-2 cells exposed to various concentrations of DHI for 12 hours and the p65 in the cytoplasm and nucleus were analyzed by western

blotting. All experiments were repeated three times with the same results.

Supplemental Figure S3. DHI does not block the nuclear translocation of p50. HeLa cells were treated with or without the indicated concentrations of DHI for 12 hours, followed by stimulation with or without TNFα (15 ng/mL) for 30 minutes. Localization of p50 was examined by immunofluorescent staining. Green signaling represents p50 staining and blue indicates nuclei stained with DAPI. Data are representative of three or more experiments with similar results.

Supplemental Figure S4. AKT inhibitor or ERK inhibitor enhances the inhibitory effect of DHI on NHL cells. (**a**) Daudi, NAMALWA, SU-DHL-4, SU-DHL-2, U2932 and OCI-Ly8 cells were exposed to various concentrations of DHI for 24 hours and the indicated proteins were detected by western blotting. (b) The effect of DHI on thermal stabilization of AKT and ERK in Daudi, SU-DHL-2 cells at various temperatures were detected by CETSA. (c) Daudi and SU-DHL-2 cells were treated for 12 hours with the indicated concentration of AKT inhibitor MK2206 or ERK inhibitor U0126, and the proteins were detected by western blotting. (d) The combination effects of DHI with the indicated inhibitor (24 hours) on proliferation of Daudi, SU-DHL-2 cells. Cell proliferation was analyzed by CCK-8 assay. Data are representative of three or more experiments with similar results. All values represent the means ± S.D. of three independent experiments. *, $P < 0.05$; **, $P < 0.01$ vs the control.

Supplemental Figure S5. DHI inhibits the stimulants-induced phosphorylation and degradation of IκBα in NHL cells. NAMALWA (**a**), Daudi and SU-DHL-2 cells (**b**) were pre-treated with DHI (0, 5, or 10 μM) for 4 hours, followed by stimulation of TNFα (15 ng/ml) for 30 minutes or LPS (20 ng/mL) for 20 minutes. Expression of p-IκBα and IκBα in the whole cell lysates were then analyzed by western blotting. All experiments were repeated three times with the same results.

Supplemental Figure S6. Ainsliadimer A alters IKKβ thermal stabilization. The effect of ainsliadimer A on thermal stabilization of IKKβ in SU-DHL-2 cells at various temperatures were analyzed by CETSA as described in materials and methods. All experiments were repeated three times with the same results.

Supplemental Figure S7. Effect of IKKα/β knockdown on the protein level of p-IκBα and IκBα. Daudi (**a**) and SU-DHL-2 (**b**) cells stably infected with NC, shIKKα, shIKKβ, or shIKKα/β were harvested. p-IκBα and IκBα were detected by western blotting. Daudi cells stimulated with 15 ng/mL TNFα for 30 minutes were used as a positive control for p-IκBα. All experiments were repeated three times with the same results.

Supplemental Figure S8. Effect of IKKβ knockdown on the distribution of phosphorylated p65 and p65 protein. Daudi and SU-DHL-2 cells that stably infected with NC, shIKKβ were harvested and the cytoplasmic fractions and nuclear

fractions were extracted, protein levels of phosphorylated p65 (p-p65) (**a**) and p65 (**b**) were detected by western blotting. The numbers at the bottom of the blots indicate the relative intensity of p-p65 protein against β-actin. All experiments were repeated three times with the same results.

Supplemental Figure S9. The effect of DHI on the mouse liver. Representative images of liver sections stained with H&E of mouse treated with vehicle or DHI. Arrow indicated infiltration of inflammatory cells and the scale bars are 10 μm.

Supplemental Figure S10. DHI reduces p52 protein level in NHL cells. (**a**) Daudi, NAMALWA, SU-DHL-4, SU-DHL-2, U2932 and OCI-Ly8 cells were treated with the indicated concentrations of DHI for 24 hours and expression of p52 were analyzed by western blotting. (**b**) Daudi and SU-DHL-2 cells were treated with various concentrations of DHI for 12 hours and the indicated proteins from the cytoplasm and nuclear fraction were detected by western blotting. All experiments were repeated three times with the same results.

Supplemental Figure S11. DHI-induced degradation of IKKα/β is independent of protein proteasome or lysosomal pathway. After pretreatment with the indicated concentration of proteasome inhibitor MG132 (**a**) or lysosomal inhibitor chloroquine (CQ) (**b**) for 30 minutes, Daudi and SU-DHL-2 cells were treated with 10 μM DHI for 12 hours and IKKα, IKKβ protein were detected by western blotting. All

experiments were repeated three times with the same results.

Supplemental Figure S12. Expression NF-κB signaling pathway components in NHL cells. The NF-κB signaling pathway relevant proteins were detected by western blotting in the indicated cells. All experiments were repeated three times with the same results.
